# GDF-15 Is Associated with Poor Physical Function in Prefrail Older Adults with Diabetes

**DOI:** 10.1155/2023/2519128

**Published:** 2023-04-28

**Authors:** Reshma Aziz Merchant, Yiong Huak Chan, Gustavo Duque

**Affiliations:** ^1^Division of Geriatric Medicine, Department of Medicine, National University Hospital, Singapore; ^2^Department of Medicine, Yong Loo Lin School of Medicine, National University of Singapore, Singapore; ^3^Biostatistics Unit, Yong Loo Lin School of Medicine, National University of Singapore, Singapore; ^4^Research Institute of the McGill University Health Centre, Montreal, Quebec, Canada; ^5^Division of Geriatric Medicine, Department of Medicine, McGill University, Montreal, Quebec, Canada

## Abstract

**Introduction:**

Growth differentiation factor 15 (GDF-15) has been shown to be a metabolic and appetite regulator in diabetes mellitus (DM) and obesity. We aimed to investigate (i) the association between GDF-15 and DM with and without poor physical function independent of inflammation and (ii) the prediction model for poor physical function in prefrail older adults.

**Methods:**

A cross-sectional study of 108-prefrail participants ≥60 years recruited for multidomain interventions. Data was collected for demographics, cognition, function, frailty, nutrition, handgrip strength (HGS), short physical performance battery (SPPB), and gait speed. Serum concentrations of GDF-15, IL-6, and TNF-*α* were measured. GDF-15 was classified into tertiles (T1, T2, and T3), and its association was studied with DM and physical function (DM poor physical function, DM no poor physical function, no DM poor physical function, and no DM no poor physical function).

**Results:**

Compared with T1, participants in T3 were significantly older, had a lower education level, had almost three times higher prevalence of DM, slower gait speed, longer chair-stand time, and lower SPPB scores. On multivariate analysis, the odds of having both DM and poor physical performance compared to having no DM and no poor physical performance were significantly higher in GDF-15 T3 vs. GDF-15 T1 (aOR 9.7, 95% CI 1.4-67.7; *p* = 0.021), and the odds of having DM no poor physical function compared to having no DM and no poor physical performance were significantly higher in GDF-15 T2 (aOR 12.7, 95% CI 1.1-143.7; *p* = 0.040) independent of BMI, IL-6, TNF-*α*, nutrition, physical function, education, age, and gender.

**Conclusion:**

The association of GDF-15 with DM-associated poor physical function is independent of inflammation in prefrail older adults. Its causal-association link needs to be determined in longitudinal studies.

## 1. Introduction

With an aging population globally, the prevalence of noncommunicable diseases (NCD) such as diabetes mellitus (DM) and frailty will continue to rise. The rising burden of type 2 DM worldwide is a major concern as it is a leading cause of adverse outcomes such as poor physical function and premature mortality. In 2017, approximately 6.3% (462 million) of the world's population was affected by type 2 DM, and 22.0% of those above 70 years old [1]. Older adults with DM are at greater risk of frailty, cardiovascular, and cerebrovascular diseases, which are common causes of declining functional ability. Countries worldwide are searching for biomarkers to identify the population at risk of NCD and subsequent disability. Aging and diabetes are both associated with low-grade inflammation and are independently associated with frailty and poor physical function [2]. Frailty is a dynamic state of the poor physiological reserve, resulting in increased vulnerability to adverse outcomes when exposed to stressors [3]. The prevalence of frailty in DM is double that of the general population and varies between 5% and 48% depending on the criteria used and the population studied [4, 5].

The burden of DM and associated complications continues to increase despite advances in clinical care and innovations in diagnosis and therapeutics [1]. Poor physical function in DM is often attributed to inflammation and to complications of diabetes, such as cerebrovascular disease, neuropathy, or retinopathy. To date, there are no validated biomarkers to predict functional complications in patients with DM. Growth differentiation factor 15 (GDF-15) is a divergent member of the transforming growth factor-beta superfamily. It is a stress-induced cytokine that has gained increasing attention recently as a biomarker of biological aging, mitochondrial dysfunction, and resilience in addition to metabolic and appetite regulators in DM and obesity [6–10]. It was first recognized in 1997 as macrophage inhibitory cytokine-1 and expressed in multiple tissues, including skeletal muscles. Apart from pregnancy, where high levels are expressed in the placenta [8], it is often found at a very low level in serum. The release of GDF-15 can be activated by various growth factors and cytokines, cellular stress, tissue injury, hypoxia, p53 activation, exercise, and drugs like metformin [7, 11]. It acts locally in an autocrine or paracrine manner as anti-inflammatory or proinflammatory, as well as through glial cell-derived neurotrophic factor family receptor alpha-like (GFRAL) receptors in the hindbrain, which are responsible for GDF-15-mediated anorexia and weight loss [12, 13]. Elevated GDF-15 has shown to have a protective effect where it promotes adaptation to systemic inflammation, improves insulin sensitivity, increases thermogenesis and lipolysis, and regulates cell regeneration, repair, and apoptosis, as well as a detrimental systemic effect where it serves as a prognostic biomarker for cardiovascular risk and cancer [6, 8, 9, 14–17]. It is possible that GDF-15 released from repeated mild mitochondrial perturbation in sepsis may offer a protective role based on mitohormesis theory and excessive release to detrimental outcomes [18].

Elevated GDF-15 is associated with incident diabetes in middle-aged adults (<60 years old) and metformin use [19, 20]. Pathologically, it is associated with metabolic diseases such as DM and cardiovascular disease, cancer, mitochondrial dysfunction, cancer cachexia, declining gait speed, and mortality [16, 21–23]. GDF-15 levels have also been shown to correlate with COVID-19 severity. It may serve as a compensatory mechanism to counteract the exaggerated immune response and can potentially be considered as a prognostic biomarker [24]. While cytokines such as interleukin-6 (IL-6) and tumour necrosis factor alpha (TNF-*α*) are known to be elevated in DM-related complications and prefrail or frail older adults, these cytokines have a limited predictive ability [2]. High GDF-15 levels have been associated with lower muscle mass and strength, frailty, slow gait speed, and declining physical function in older adults [21, 22, 25, 26]. There are no studies on the association of GDF-15 with physical function in prefrail older adults with DM. We aimed to investigate (i) the association between GDF-15 and DM with and without poor physical function independent of inflammation in prefrail older adults and (ii) the prediction model for poor physical function in prefrail older adults.

## 2. Materials and Methods

This was a cross-sectional study of 108 community-dwelling prefrail participants ≥60 years recruited for multidomain interventions from two primary care settings and the community. Inclusion criteria included prefrail participants who could provide consent and follow instructions. Exclusion criteria included nursing home residents, bedbound, or chairbound. The study methodology is explained in a prior study, and only participants who agreed on blood taking are included in the current analysis [27].

### 2.1. Demographics and Covariates

Interview questionnaires were administered by trained research staff on demographics, chronic diseases, medications, physical function, cognition, frailty, depression, and perceived health. Frailty was assessed using the FRAIL scale (fatigue, resistance, aerobic, illness, and loss of weight), and prefrailty was defined by a score of 1-2 with a maximum score of 5 [28]. Cognition was assessed using the Montreal Cognitive Assessment (MoCA) score [29]. Activities of daily living (ADL) were evaluated using the Katz ADL scale and instrumental activities of daily living (IADL) using Lawton's IADL scale [30, 31]. Physical activity was assessed using the Rapid Assessment of Physical Activity (RAPA) tool with a maximum score of 6, and physically active was defined by scores between 5 and 6 on the RAPA scale [32]. A fifteen-item Geriatric Depression Scale (GDS) was used to evaluate depression where a score of >5 was classified as depressed [33]. Nutrition was assessed using the Nutritional Assessment Short-Form (MNA-SF) tool with a maximal score of 14 [34]. Perceived health was evaluated using the EuroQol vertical visual analogue scale [35].

Physical function tests included assessment of handgrip strength (HGS), gait speed, and the Short Physical Performance Battery (SPPB) test. Maximum HGS of the dominant hand was measured using a Jamar hand dynamometer in a seated position with the elbow flexed at 90°. Low HGS was defined based on the 2019 Asian Working Group for Sarcopenia criteria with cutoffs of 28 kg for males and 18 kg for females [36]. Gait speed was measured over 4 meters. Slow gait speed was defined as <1.0 m/s. SPPB (3 domains—gait speed, balance, and 5-times chair stand time) was measured with a maximal score of 12, with 4 points per domain. Participants were classified as having poor physical function if they had a low HGS, slow gait speed, or SPPB total score ≤ 9 [36]. The TNF-*α*, IL-6, and GDF-15 cytokines were measured by the accredited hospital-based laboratory. The TNF-*α* cytokine was measured by immunoenzymetric assays with a detection range between 1.0 and 498 pg./mL. IL-6 was measured using the electrochemiluminescence immunoassay (ECLIA) with a detection range between 1.5 and 50,000 pg./mL and GDF-15 using the enzyme-linked immunosorbent assay with a detection range of 2.0-2400 pg./mL. GDF-15, IL-6, and TNF-*α* were classified into tertiles (T1, T2, and T3). Tertile instead of median cutoff was used based on the mitohormesis theory, where GDF-15 released in response to mild mitochondrial stress may be protective and excessive release contributes to poor outcomes, as shown in Supplementary Figure 1 where there was no significant difference between SPPB scores in GDF-15 T1 and T2. The associations of GDF-15 tertiles with DM and physical function (DM poor physical function, DM no poor physical function, no DM poor physical function, and no DM no poor physical function) were analyzed.

### 2.2. Statistics

The IBM SPSS statistics software, version 28, was used for data analysis with statistical significance set at a 2-sided *p* value of 0.05. Data were presented as mean ± standard deviation (SD) for continuous normally distributed variables; otherwise, median (interquartile range) and number (%) for categorical data were presented. Associations of GDF-15 tertiles with categorical data were assessed using the chi-square test and one-way ANOVA for numerical variables with Bonferroni's correction for pairwise comparisons. Multinomial regression analysis was performed to determine the association between GDF-15 tertiles and DM with and without poor physical function adjusted for gender, age, BMI, education, RAPA, nutrition, and inflammation (IL-6 and TNF-*α*). Odds ratios (ORs) with 95% confidence intervals (CIs) were presented. A prediction model using the b-estimates of the GDF-15 tertiles, TNF-*α* tertiles, IL-6 tertiles, diabetes, age, years of education, BMI, gender, RAPA, and total MNA-SF on poor physical function was developed, and receiver operating characteristics (ROC) were performed to evaluate its discriminant capability.

### 2.3. Ethics Approval and Informed Consent

Ethics approval was obtained from the National Healthcare Group Domain Specific Review Board (Reference: 2017/00035 and 2018/01183). Informed consent was obtained from all participants.

## 3. Results

One hundred and eight participants ≥60 years old who participated in the multidomain interventions had complete biomarker data available. Demographics, perceived health, and functional measures were stratified according to GDF-15 tertiles (Table 1). Participants in T1 were significantly younger (69.2 ± 4.6 years) compared with tertile 2 (73.6 ± 7.1 years). Participants in T3 had a significantly lower education level compared with T2 and T1 (6.7 ± 3.2, 9.4 ± 5.1, and 10.6 ± 4.7 years, respectively). Almost two-thirds of those in T3 had diabetes compared with one-third in T2 and one-fifth in T1. There were significant differences between GDF-15 tertiles for gait speed, SPPB, and 5× STS time. T3 had a significantly slower gait speed (median [IQR] 0.9 [0.4] m/s) compared with T1 (median [IQR] 1.1 [0.3] m/s). Similarly, T3 had significantly lower SPPB (median [IQR] 9.0 [3.0]) compared with T2 (median [IQR] 11.0 [2.0]) and T1 (median [IQR] 11.0 [2.0]). Five times chair-stand times were significantly longer in T3 compared with T2 and T1, at 15.1 ± 4.3 s, 11.6 ± 3.5 s, and 12.4 ± 2.5 s, respectively. While not significant, the trend for poor physical function prevalence increased with increasing tertiles, from 57.1% in T1 to 81.1% in T3.

**Figure 1:**
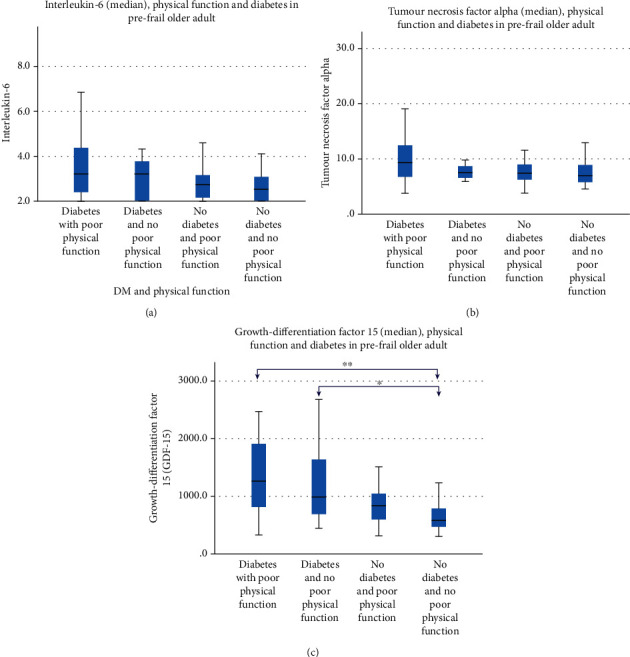
(a) Median interleukin-6 levels, (b) median tumour necrosis alpha levels, and (c) median growth differentiated 15 levels in diabetics and nondiabetics with poor physical function.

DM poor physical function compared with DM no poor physical function group had significantly higher median GDF-15 levels but not IL-6 and TNF-*α* (Figure 1). Amongst the DM poor physical function group, 60.6% belonged to GDF-15 T3 compared with 36.4% of the DM no poor physical function and only 15.8% of the no DM no poor physical function (Table 2). On multivariate analysis, the odds of having both DM and poor physical performance compared to having no DM and no poor physical performance were significantly higher in GDF-15 T3 vs. GDF-15 T1 (aOR 9.7, 95% CI 1.4-67.7; *p* = 0.021), and the odds of having DM no poor physical function compared to having no DM and no poor physical performance were significantly higher in GDF-15 T2 (aOR 12.7, 95% CI 1.1-143.7; *p* = 0.040) independent of BMI, IL-6, TNF-*α*, nutrition, physical function, education, age, and gender.

### 3.1. Prediction Model for Poor Physical Function

**Figure 2:**
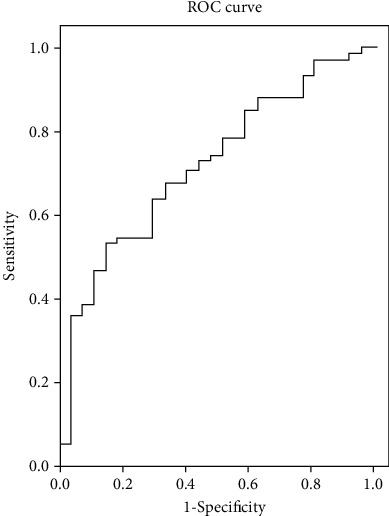
Receiver operating characteristics (ROC) analysis.

A logistic regression was performed on poor physical function using GDF-15 tertiles, TNF tertiles, IL-6 tertiles, diabetes, age, years of education, BMI, gender, RAPA, and total MNA-SF, and the B-estimates were used as the weighted scores to develop the prediction model (Table 3), with an AUC of 0.719, 95% CI (0.612–0.826), *p* = 0.001 (Figure 2).  Table 4 shows the discriminant capability of the scores from the prediction model. Subjects in quartiles 3 and 4 of the prediction scores had a positive predictive value of 73% and 96% of being at risk for having a poor physical function. Variables significantly associated with poor physical performance (quartile 4) were age, education, physical activity, GDF-15, IL-6, and TNF-*α* but not DM (Table 5).

## 4. Discussion

Our study showed that the highest tertile of GDF-15 level in serum was significantly associated with DM poor physical function but not with the no DM poor physical function group after adjustment for inflammation, BMI, and nutrition. Two-thirds of those in the highest GDF-15 tertile had underlying DM, and more than three-quarters had poor physical function. The median level of GDF-15 was significantly higher in the DM poor physical function group but not IL-6 or TNF-*α*. While the findings of elevated levels of GDF-15 in DM are not new, and GDF-15 levels are also known to increase with metformin use, the significant association with the DM poor physical function group independent of IL-6, TNF-*α*, and nutrition indicates that it may serve as a potential biomarker for prefrail older adults with DM and poor physical function [20]. Based on our prediction model, higher tertiles of IL-6, TNF-*α*, and GDF-15 were significantly associated with poor physical function in general but not in DM suggesting a role for other underlying mechanisms in the DM poor physical function group such as mitochondrial dysfunction. The association of GDF-15 with physical performance, such as gait speed, has been shown in multiple studies, but no studies have specifically studied poor physical function in persons with diabetes [21, 22]. The association of muscle function, strength, and mass with GDF-15 has shown mixed results depending on the age and population studied. Oba et al. reported a significant association with low muscle strength and lower extremity function in older adults with cardiometabolic disease who visited a frailty clinic, whereas Semba et al. reported no association with HGS but a significant association with walking speed and lower physical performance [20, 22]. The prevalence of low HGS did not differ amongst the different GDF-15 tertiles in our study population.

GDF-15 is expressed in most organs, including the bladder, kidney, colon, stomach, liver, gall bladder, pancreas, endometrium, and muscles, and cell types including cardiomyocytes, adipocytes, macrophages, endothelial, myocytes, and vascular smooth muscle cells [7]. GDF-15 is regarded as a mitokine and a known biomarker for mitochondrial dysfunction, where it is found to be elevated in various myopathies [8, 23, 37]. Aging, obesity, and DM are associated with mitochondrial dysfunction, especially in skeletal muscle [38]. Mitochondrial function is a crucial determinant of fuel homeostasis, inflammation, immunity, and apoptosis [37]. Mitochondrial dysfunction contributes to many diseases, but mitochondrial perturbations may be beneficial in activating the innate immune response during cellular stress, a phenomenon called mitohormesis [18]. It is still unknown if GDF-15 released in response to repeated mitochondrial stress of mild intensity confers a protective role in restoring cell function and longevity [18]. In mice, elevated GDF-15 has shown to be protective against diet-induced obesity and insulin resistance in the context of selective muscle mitochondrial dysfunction [39]. While the secretion of a small amount of GDF-15 may be necessary to correct and restore some aspects of mitochondrial dysfunction in mitohormesis, uncontrolled secretions can contribute to the aging process and disease progression [18, 40]. Our study highlights that the mechanism of action of GDF-15 and its role in poor physical function in DM may be independent of inflammation. Having very high GDF-15 (T3) levels in our study participants was independently associated with an elevated likelihood of having both DM and poor physical function and high GDF-15 (T2) with having DM no poor physical function when no DM no poor physical function was used as reference requires further validation in larger prospective studies taking into account the protective role of metformin which can also be associated with elevated GDF-15 levels [6].

In recent years, there has been increasing literature on the role of GDF-15 as a prognostic biomarker in the acute care setting, including sepsis, renal disease, chronic obstructive airway disease, COVID-19, and myocardial infarction [23, 24, 41, 42]. The pathological mechanism of sepsis in humans may be mediated through the mitochondrial stress response where it induces the release of distinct secretory proteins from cells including GDF-15. In sepsis, a rise in GDF-15 may play a role in metabolic adaptation and organ protection [41]. There was increased mortality demonstrated in the lipopolysaccharide and polyinosinic polycytidylic acid mouse models injected with antibody-targeting GDF-15, possibly related to tissue damage caused by excessive inflammation. In the same mouse models, inhibitions of GDF-15 led to increased cardiac troponin I with significantly decreased left ventricular stroke volume, and significantly raised blood urea and creatinine [41]. On the contrary, several other studies showed an overall poor prognosis in patients with elevated GDF-15 levels on admission [26]. Elevated GDF-15 levels on admission to the hospital have been associated with poor recovery, slowing of gait speed, and declining nutrition status at 30 days which may partly be explained by reduced physical function in the hospitalized group [26]. Increased circulating GDF-15 is significantly associated with significant cardiovascular events in patients with coronary artery disease, major bleeding in patients receiving antithrombotic therapies, chronic kidney diseases, and cancers [14].

The strength of our study includes a detailed evaluation of physical function and community-dwelling prefrail older adults presumably free of acute illness or sepsis. However, several limitations warrant mention. First, the small sample size and data only on prefrail only which may limit generalizability at the population level. Second, inflammatory biomarker tertiles were selected for analysis based on the mitohormesis theory, resulting in a very small sample size for certain groups. Nonetheless, the results did show a significant difference which may help pave the path for future research. Analysis based on median cutoffs (Supplementary Table 1) did not show a significant association. Third, due to the cross-sectional nature of the study, the cause-effect relationship remains unclear, and it is not known if GDF-15 is elevated due to cellular stress or as a response to cellular stress. Fourth, we had no information on the duration of diabetes, complications, or metformin use. However, Oba et al. showed a significant association of GDF-15 with physical function even after adjustment for metformin use [20]. Last, DM was based on self-report and subject to recall bias.

There are still significant gaps in the molecular knowledge of GDF-signaling in both healthy and at-risk older adults [15]. It is not known if GDF-15 is elevated due to cellular stress in those with poor physical function, mitochondrial dysfunction, or bystander marker for other underlying metabolic disorders [43]. GDF-15 shows great promise to be a biomarker for disease prediction and prognosis, identifying at-risk groups who may benefit from intensive intervention and the potential application of GDF-15-based therapies in cancer cachexia, DM, and obesity. There seems to be a dose-response effect depending on the outcome of interest, and the knowledge of the mechanism of action for different GDF-15 thresholds in high-risk older populations is an area for future research. In addition, the impact of very high GDF-15 levels in frail, sarcopenic, or nonobese individuals on appetite with subsequent weight loss and decline in physical function needs to be validated in a larger group.

## 5. Conclusion

GDF-15 was significantly elevated in the DM poor physical function group. The association of GDF-15 with DM poor physical function was independent of inflammation and nutrition status in prefrail older adults. It is not known if increased levels of GDF-15 are the cause or consequence of poor physical function in persons with DM. Future prospective longitudinal studies are required to evaluate the potential role of GDF-15 in screening persons with diabetes at risk of poor physical function and the impact of multidomain interventions in reducing the long-term risk of poor physical function.

## Figures and Tables

**Table 1:** Characteristics of subjects by GDF-15 tertiles.

	All *n* = 108	Tertile 1 *n* = 35 (30.9)	Tertile 2 *n* = 36 (34.6)	Tertile 3 *n* = 37 (34.6)	*p* value
Age	71.7 ± 5.8	69.2 ± 4.6^a^	73.6 ± 7.1^a^	72.2 ± 4.5	0.004
Gender^#^					0.240
Male	43 (39.8)	10 (23.3)	17 (39.5)	16 (37.2)	
Female	65 (60.2)	25 (38.5)^a^	19 (29.2)	21 (32.2)	
Ethnicity^#^					0.682
Chinese	91 (84.0)	30 (33.0)	31 (34.0)	30 (33.0)	
Malay	4 (3.6)	2 (50)	0 (30.8)	2 (50.0)	
Indian	13 (12.4)	3 (23.0)	5 (38.5)	5 (38.5)	
BMI (kg/m^2^)	25.3 ± 4.0	25.1 ± 3.9	25.0 ± 3.2	25.8 ± 4.8	0.657
Employment^∗^					0.024
Working	30 (30.6))	38 (37.1)	10 (27.7)	7 (18.9)	
Unemployed	13 (13.3)	5 (14.3)	5 (13.9)	3 (8.1)	
Retired	50 (51.0)	16 (45.7)	20 (55.6)	24 (64.9)	
Homemaker	5 (5.1)	1 (2.9)	1 (2.8)	3 (8.1)	
Education (years)	8.8 ± 4.6	10.6 ± 4.7^a^	9.4 ± 5.1^b^	6.7 ± 3.2^a,b^	<0.001
Diabetes	44 (40.7)	8 (22.9)	12 (33.3)	24 (64.9)	<0.001
Hyperlipidaemia	80 (74.8)	26 (74.3)	26 (74.3)	28 (75.7)	0.988
Hypertension	63 (58.9)	18 (51.4)	19 (54.3)	26 (70.3)	0.213
Nutrition (median [IQR])	14.0 [2.0]	14.0 [2.0]	13.0 [1.0]	14.0 [2.0]	0.238
≥5% weight loss in past year	7 (6.5)	2 (5.7)	1 (2.8)	4 (10.8)	0.373
≥1 ADL impairment	20 (18.5)	3 (8.6)	6 (16.7)	11 (29.7)	0.065
≥1 IADL impairment	56 (29.3)	4 (11.4)	6 (16.7)	12 (32.4)	0.069
Perceived health (EQ-VAS)	68.8 ± 12.6	69.7 ± 12.0	69.4 ± 11.2	67.2 ± 14.4	0.637
Physically active	19 (17.6)	9 (25.7)	7 (19.4)	3 (8.1)	0.137
Depression	21 (19.4)	6 (17.1)	5 (13.9)	10 (27.0)	0.335
MoCA (median [IQR])	27.7 [4.0]	28.0 [3.0]	27.0 [4.0]	27.5 [5.0]	0.275
Sarcopenia (AWGS 2019)	19 (19.0))	6 (17.6)	6 (18.2)	7 (21.2)	0.923
Gait speed (m/s) (median [IQR])	1.0 [0.5]	1.1 [0.3]^a^	1.0 [0.4]	0.9 [0.4]^a^	0.021
Low gait speed	50 (46.7)	10 (28.6)	19 (54.3)	21 (56.8)	0.031
5× chair-stand time (sec)	13.1 ± 3.8	12.4 ± 2.5^a^	11.6 ± 3.5^b^	15.1 ± 4.3^a,b^	<0.01
SPPB score (median [IQR])	11.0 [3.0]	11.0 [2]^a^	11.0 [2]^b^	9.0 [3]^a,b^	0.012
SPPB ≤ 9.0	34 (31.5)	6 (17.1)	8 (22.2)	20 (54.1)	0.001
HGS (kg) (median [IQR])	20.3 [10.1]	19.2 [9.6]	20.6 [9.3]	19.9 [13.5]	0.779
Low handgrip	58 (54.2)	16 (45.7)	22 (61.1)	20 (55.6)	0.420
Poor physical function	78 (72.2)	20 (57.1)	28 (77.8)	30 (81.1)	0.051

^∗^ *n* = 98; mean ± SD; SPPB: short physical performance battery; HGS: handgrip strength; MoCA: Montreal Cognitive Assessment; ADL: activity of daily living; IADL: instrumental activity of daily living.

**Table 2:** Association of GDF-15 tertiles with diabetes and physical function.

Diabetes	Poor physical function	*N*	GDF tertiles; *n* (%)			Tertile 2 vs. Tertile 1		Tertile 3 vs. Tertile 1	
			Tertile 1	Tertile 2	Tertile 3				
						Unadjusted	Adjusted^#^	Unadjusted	Adjusted^#^
						OR (95% CI) *p* value	OR (95% CI) *p* value	OR (95% CI) *p* value	OR (95% CI) *p* value
No	No	19	13 (68.4)	3 (15.8)	3 (15.8)	Reference			
No	Yes	45	14 (31.1)	21 (46.7)	10 (22.2)	6.5 (1.6-27.1) *p* = 0.010	4.7 (0.9-25.2) *p* = 0.070	3.1 (0.7-13.8) *p* = 0.138	1.8 (0.3-11.1) *p* = 0.508
Yes	No	11	2 (18.2)	5 (45.5)	4 (36.4)	10.8 (1.4-85.4) *p* = 0.024	12.7 (1.1-143.7) *p* = 0.040	8.7 (1.05-71.6) *p* = 0.045	9.6 (0.6-141.5) *p* = 0.101
Yes	Yes	33	6 (18.2)	7 (21.2)	20 (60.6)	5.1 (0.96-26.7) 0.056	2.8 (0.4-19.5) *p* = 0.308	14.4 (3.1-68.2) *p* = 0.001	9.7 (1.4-67.7) *p* = 0.021

#Adjusted for age, years of education, BMI, RAPA, total MNA-SF score, IL-6 tertiles, and TNF-*α* tertiles. Values are *n* (%).

**Table 3:** Logistic regression weights on the variables for the prediction model for poor physical function.

Variable	Weights from the B estimate
Diabetes	-0.092
Age	0.093
BMI	0.032
Education years	0.041
Total MNA score	0.047
RAPA score	-0.163
Male	-0.059
GDF-15 tertile 2	0.513
GDF-15 tertile 3	0.886
TNF-*α* tertile 2	0.384
TNF-*α* tertile 3	0.146
IL-6 tertile 2	0.627
IL-6 tertile 3	0.503
Constant	-7.705
AUC	0.719, 95% CI (0.612–0.826) *p* = 0.001

BMI: body mass index; IL-6: interleukin-6; TNF-*α*: tumour necrosis factor alpha; GDF-15: growth differentiation factor 15.

**Table 4:** Prediction risk of poor physical function.

Prediction score	Poor physical function		*p* value
	No (*n* = 27)	Yes (*n* = 75)	
Total score			
Mean ± SD	0.70 ± 0.77	1.32 ± 0.80	0.001
Range	-0.86 to 2.44	-0.66 to 3.34	
Quartiles (Q1 to Q4)			
Q1: Up to 0.5	11 (44.0)	14 (56.0)	0.006
Q2: >0.5 to 1.1	8 (34.8)	15 (65.2)	
Q3: >1.1 to 1.7	7 (26.9)	19 (73.1)	
Q4: >1.7	1 (3.6)	27 (96.4)	

Values are *n* (row %).

**Table 5:** Characteristics of the 4 quartiles of the Prediction Model.

Variable	Quartile 1	Quartile 2	Quartile 3	Quartile 4	*p* value
Age	67.4 ± 3.4	70.1 ± 4.4	72.0 ± 3.5	77.2 ± 5.8	<0.001
BMI	23.7 ± 3.5	25.8 ± 4.3	25.7 ± 3.7	25.9 ± 4.5	0.182
Education years	11.7 ± 4.4	9.2 ± 4.6	7.7 ± 4.4	7.1 ± 4.3	0.001
Total MNA-SF score	12.7 ± 1.5	13.1 ± 1.1	13.1 ± 1.7	13.0 ± 1.4	0.713
RAPA score	4.2 ± 1.8	3.6 ± 1.4	3.0 ± 1.1	2.8 ± 1.3	0.001
Gender					0.565
Female	18 (29.0)	14 (22.6)	16 (24.2)	15 (24.2)	
Male	7 (17.5)	9 (22.5)	11 (27.5)	13 (32.5)	
Diabetes					0.318
No	18 (28.6)	16 (25.4)	15 (23.8)	14 (22.2)	
Yes	7 (17.9)	7 (17.9)	11 (28.2)	14 (35.9)	
GDF-15					<0.001
Tertile 1	21 (61.8)	9 (26.5)	4 (11.8)	0 (0.0)	
Tertile 2	2 (5.7)	10 (28.6)	10 (28.6)	13 (37.1)	
Tertile 3	2 (6.1)	4 (12.1)	12 (36.4)	15 (45.5)	
TNF-*α*					0.005
Tertile 1	16 (43.2)	9 (24.3)	9 (24.3)	3 (8.1)	
Tertile 2	7 (20.6)	6 (17.6)	9 (26.5)	12 (35.3)	
Tertile 3	2 (6.5)	8 (25.8)	8 (25.8)	13 (41.9)	
IL-6					<0.001
Tertile1	18 (50.0)	9 (25.0)	6 (16.7)	3 (8.3)	
Tertile 2	4 (10.5)	9 (23.7)	10 (26.3)	15 (39.5)	
Tertile 3	3 (10.7)	5 (17.9)	10 (35.7)	10 (35.7)	

Values are *n* (row %), mean ± SD.

## Data Availability

Data is available on request.

## Supplementary Materials

Supplementary Materials Supplementary Table 1 shows the association of the median GDF-15 with diabetes and physical function which was not significant. Supplementary Figure 1 supports the mitohormesis theory, where SPPB changes in tertiles 1 and 2 were not significant but significant when tertiles 1 or 2 were compared with tertile 3.
