# Supplementary material for: GDF-15 Is Associated with Poor Physical Function in Prefrail Older Adults with Diabetes
Source: J Diabetes Res. 2023 Apr 28;2023:2519128. doi: 10.1155/2023/2519128 (PMC10162869; doi:10.1155/2023/2519128)

Supplementary Table 1. Association of Median GDF with Diabetes and Physical Function

| Diabetes | Poor Physical Function | N | GDF-15 < 878.5  (n = 54) | GDF-15 ≥ 878.5  (n = 54) | Unadjusted  OR (95% CI)  p-value | Adjusted^#^  OR (95% CI)  p-value |
| --- | --- | --- | --- | --- | --- | --- |
| No | No | 19 | 14 (73.7) | 5 (26.3) | Reference | |
| No | Yes | 45 | 24 (53.3) | 21 (46.7) | 2.5 (0.8-8.0)  p = 0.136 | 1.7 (0.5-6.1)  p = 0.432 |
| Yes | No | 11 | 5 (45.5) | 6 (54.6) | 3.4 (0.7-16.0)  p = 0.129 | 2.4 (0.4-15.2)  p = 0.368 |
| Yes | Yes | 33 | 11 (33.3) | 22 (66.7) | **5.6 (1.6-19.6)**  **0.007** | 3.3 (0.8-13.2)  p = 0.098 |

# Adjusted for age, years of education, BMI, RAPA, Total MNA-SF score, IL-6 Tertiles, TNF-α Tertiles.

Values are n (%). OR: odds ratio. GDF-15: Growth Differentiation Factor 15.

Supplementary Figure 1. SPPB and GDF-15 Tertiles in A: Diabetics and B: Non-diabetics


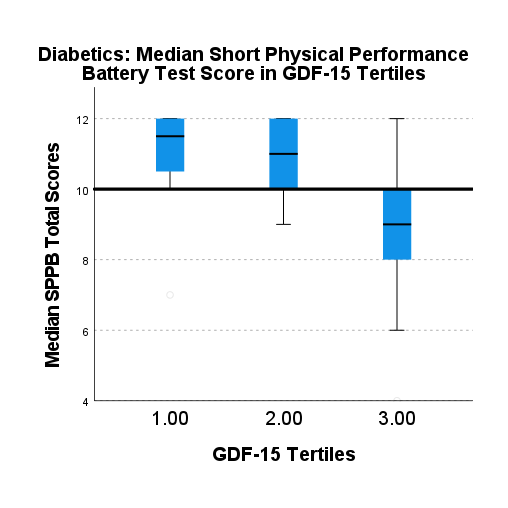


*

*


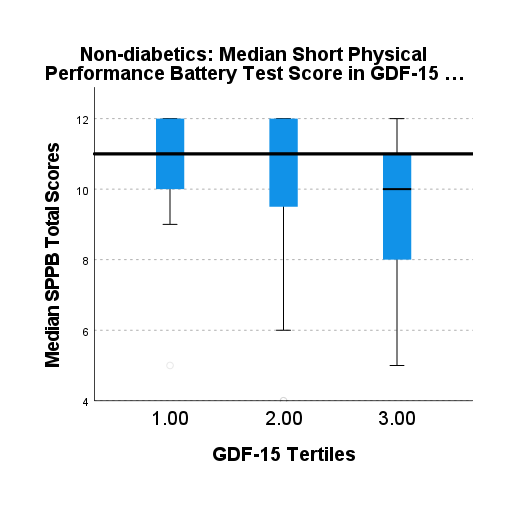

Supplement: Supplementary Materials — Supplementary Table 1 shows the association of the median GDF-15 with diabetes and physical function which was not significant. Supplementary Figure 1 supports the mitohormesis theory, where SPPB changes in tertiles 1 and 2 were not significant but significant when tertiles 1 or 2 were compared with tertile 3. [file 2519128.f1.docx]
